# FeCoNi Sulfides Derived From *In situ* Sulfurization of Precursor Oxides as Oxygen Evolution Reaction Catalyst

**DOI:** 10.3389/fchem.2020.00334

**Published:** 2020-05-05

**Authors:** Wanqing Teng, Mengtian Huo, Zhaomei Sun, Wenrong Yang, Xiangjiang Zheng, Caifeng Ding, Shusheng Zhang

**Affiliations:** ^1^Shandong Provincial Key Laboratory of Detection Technology for Tumor Markers, College of Chemistry and Chemical Engineering, Linyi University, Linyi, China; ^2^Key Laboratory of Optic-Electric Sensing and Analytical Chemistry for Life Science, Ministry of Education, College of Chemistry and Molecular Engineering, Qingdao University of Science and Technology, Qingdao, China

**Keywords:** oxygen evolution reaction, electrocatalyst, FeCoNi sulfides, nanowires array, *in situ* sulfurization

## Abstract

It is highly promising to design and develop efficient and economical electrocatalysts for oxygen evolution reaction (OER) in alkaline solution. In this work, we prepare FeCoNi sulfide composites (including FeS, Co_3_S_4_, and Ni_3_S_4_) derived from *in situ* sulfurization of precursor oxides on carbon cloth (CC), which are used to become an OER catalyst. Such catalyst shows excellent OER performance, low overpotential, small Tafel slope, and high electrochemical stability, and it is a promising electrocatalyst for OER in alkaline media.

## Introduction

The excessive consumption of fossil energy and the resulting serious environmental problems have triggered strong demand for renewable alternative energy (Chow et al., 2003; Zheng et al., 2014). Hydrogen energy is regarded as a clean and ideal energy carrier that could replace fossil energy (Dresselhaus and Thomas, 2001; Zheng et al., 2017, 2018b). Electrochemical water splitting provides us a promising strategy to largely produce hydrogen (Turner, 2004; Lu et al., 2017). However, hydrogen evolution is seriously restricted by anodic water oxidation due to the multi-electron transfer process and high activation energy barrier (Yin et al., 2010; Yang et al., 2017; Ke et al., 2018; Zheng et al., 2018a; Tang et al., 2019). Therefore, efficient catalysts to reduce activation energy should be developed to boost the water oxidation process. Noble metal oxides (RuO_2_ and IrO_2_) exhibit excellent catalytic characters in oxygen evolution reaction (OER), but their widespread applications are limited due to scarce resources and high costs (Lee et al., 2012; Reier et al., 2012). Hence, it is necessary to develop efficient and economical OER electrocatalysts.

In recent years, transition-metal oxides and hydroxyl oxide have attracted great interest for catalysts (Lu et al., 2016; Guo et al., 2017; Zhang et al., 2017; Jin et al., 2018; Zhao et al., 2018). Specifically, ferric oxyhydroxide (FeOOH) has shown efficient activity for the OER process (Chemelewski et al., 2014; Luo et al., 2017; Park et al., 2017). Regardless of its abundant reserves and low cost, its performance for OER has certain disparities in comparing with the noble metal catalysts. Many ways have been taken to improve catalysis performance, such as enhancing the conductivity of materials, increasing the specific surface area of materials, doping heteroatom modification, and so on (Feng et al., 2016a,b; Kuang et al., 2017; Li F. et al., 2018). Research shows that transition metal sulfides have better oxygen evolution catalysis performance than oxides because transit metal sulfides have diverse element composition, controllable electronic structure, and fast charge transfer speed (Liu et al., 2016; Chai et al., 2018; Li H. et al., 2018; Zhang et al., 2018).

In this manuscript, we design and develop FeCoNiS sulfides derived from *in situ* sulfurization of precursor oxides on carbon cloth (CC) through two-step hydrothermal methods. At first, FeCoNi-FeOOH nanoarray on CC is prepared through hydrothermal method. Secondly FeCoNiS sulfides derived from *in-situ* sulfurization is prepared through the second hydrothermal method. It shows excellent OER activity needing overpotentials of 220.5 and 269.9 mV to attain current densities of 10 and 100 mA cm^−2^ in 1.0 M KOH. It is a promising electrocatalyst for OER in alkaline media.

## Results and Discussions

**Figure 1:**
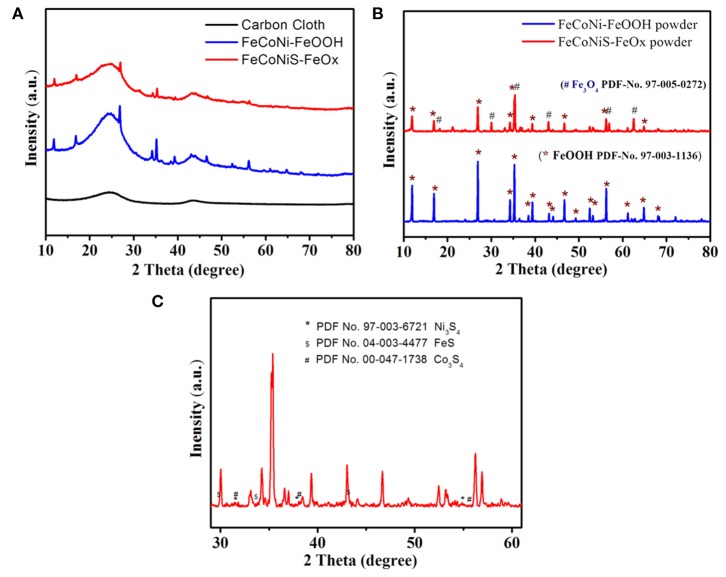
**(A)** X-ray diffraction (XRD) patterns of different oxygen evolution reaction (OER) catalysts. **(B)** XRD patterns comparison of different catalysts powder. **(C)** XRD pattern of the product after sulfidation treatment.

X-ray diffraction (XRD) patterns of these catalysts are shown in Figure 1A. There are two broad diffraction peaks on the bottom curve, which are the amorphous peaks of CC. The middle curve is the XRD pattern of the precursor oxides without sulfide treatment. The peaks at 11.95, 16.87, 26.91, 35.26, 46.69, 56.21, and 64.78° can be indexed to the (110), (200), (310), (211), (411), (521), and (541) planes of FeOOH phase (PDF No. 97-003-1136). The XRD pattern of the product after sulfide treatment is on the top. The diffraction peak intensity is obviously lower than that of the middle curve. For better structural analysis of the product, the powder of the precursor oxides and sulfide products scrapped from CC are characterized by XRD again. XRD curves are shown in Figures 1B,C. The main component of precursor oxides is still FeOOH. Considered that Co and Ni are in the precursor, we name the precursor as FeCoNi-FeOOH. The XRD curve of sulfide product shows that there are new phases, including Fe_3_O_4_ (PDF No.97-005-0272), FeS (PDF No. 04-003-4477), Ni_3_S_4_ (PDF No. 97-003-6721), and Co_3_S_4_ (PDF No. 00-047-1738). Part of FeOOH is reduced to Fe_3_O_4_, so the product is named FeCoNiS-FeO_x_.

**Figure 2:**
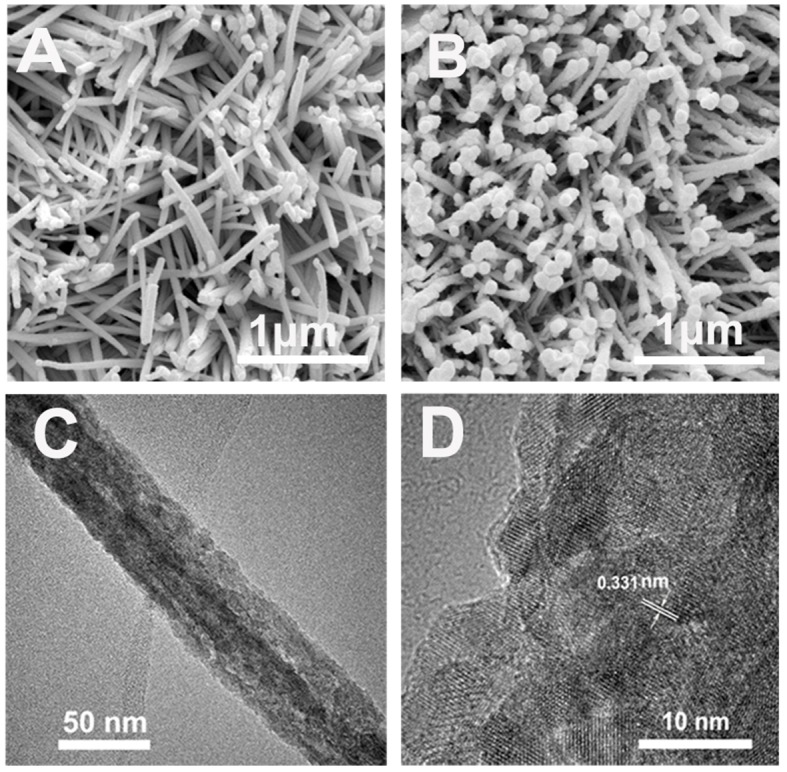
**(A)** SEM pattern of precursor (FeCoNi-FeOOH). **(B)** SEM pattern of FeCoNiS-FeO_x_. **(C)** TEM pattern of FeCoNiS-FeO_x_. **(D)** HRTEM pattern of FeCoNiS-FeO_x_.

Scanning electronic microscopy (SEM) pattern of the precursor (FeCoNi-FeOOH) is shown in Figure 2A. There are specific and uniform nanowires array on the surface of CC. SEM pattern of FeCoNiS-FeO_x_ is shown in Figure 2B. Obviously, the precursor (FeCoNi-FeOOH) nanowires are smooth, and the product (FeCoNiS-FeO_x_) is relatively rough. This means that the product has structural change after sulfidation, which corresponded to the XRD patterns in Figures 1B,C. The catalyst nanowire feature is also shown in transmission electron microscopy (TEM) characterization (Figure 2C). Image taken from the product shows about 50-nm-thick nanowires. High-resolution TEM (HRTEM) reveals that the product is highly crystallized with well-resolved lattice fringes (Figure 2D). The interplanar spacing of 0.331 nm could be assigned to the (310) plane of FeOOH.

**Figure 3:**
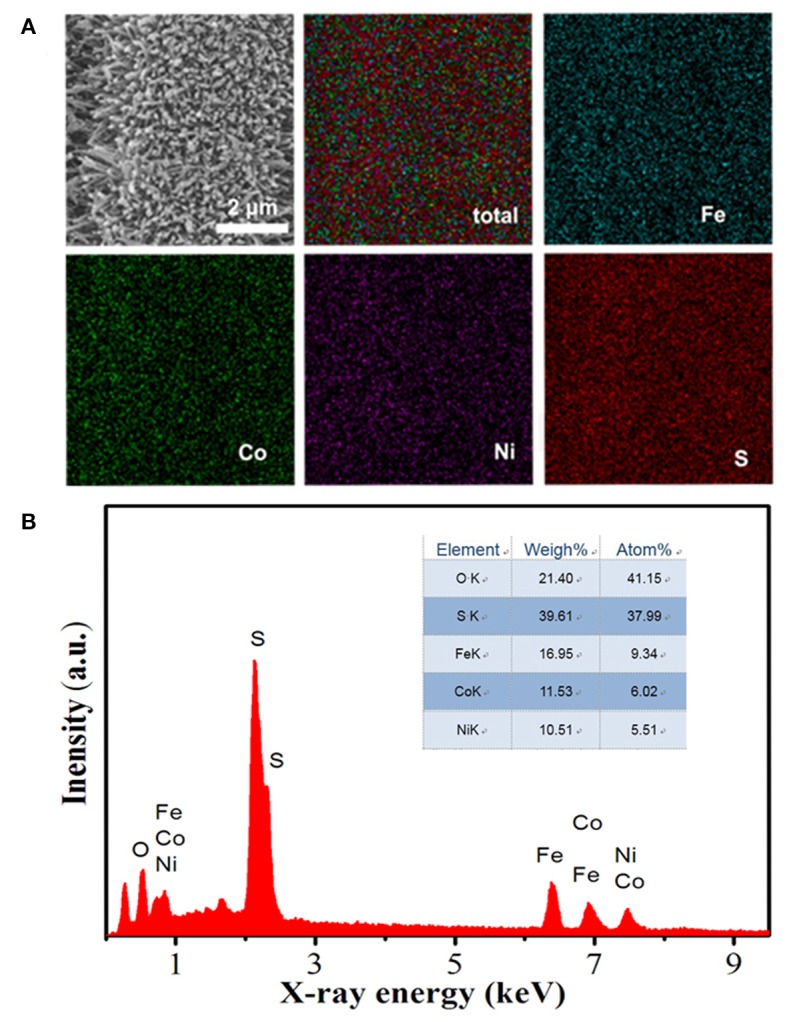
**(A)** Energy-dispersive X-ray (EDX) mapping images of FeCoNiS-FeOx. **(B)** EDX element distribution pattern.

The corresponding energy-dispersive X-ray (EDX) elemental mapping images of FeCoNiS-FeO_x_ are shown in Figure 3A, which demonstrate unique distribution of Fe, Co, Ni, and S elements. EDX pattern is shown in Figure 3B, which exhibits types and relative amounts of different elements based on the position and intensity of element spectral lines.

**Figure 4:**
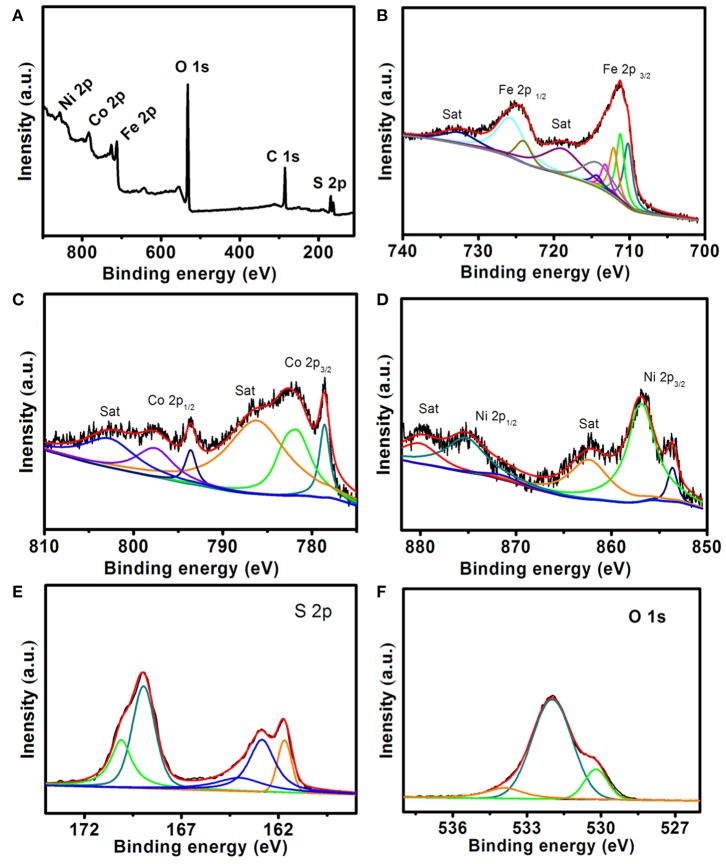
X-ray photoelectron spectroscopy (XPS) spectra of FeCoNiS-FeO_x_ catalyst. **(A)** Survey spectrum. **(B)** Fe 2p. **(C)** Co 2p. **(D)** Ni 2p. **(E)** S 2p. **(F)** O 1s.

X-ray photoelectron spectroscopy (XPS) of FeCoNiS-FeOOH is shown in Figure 4, which was performed to characterize the chemical states of different elements. Figure 4A is full-scale XPS spectrum, further revealing the presences of Fe, Co, Ni, S, and O elements in the catalyst. As shown in Figure 4B, high-spin Fe^3+^ of FeOOH contains unpaired electrons and therefore exhibit multiplet structures in Fe 2p_3/2_ area. The characteristic peaks of Fe 2p_1/2_ is at 725.8 eV. The satellite peaks (identified as “Sat.”) are at 719.3 and 732.3 eV, which are relevant to Fe 2p_3/2_ and Fe 2p_1/2_ of FeOOH (Biesinger et al., 2011; Zeng et al., 2012; Zhou et al., 2018). There are two peaks at 714.4 and 723.9 eV, which are relevant to Fe_3_O_4_. In Figure 4C, there exhibit two spin-orbit doublets. The first doublet is at 778.6 and 793.5 eV, assigned to Co 2p_3/2_ and Co 2p_1/2_ of Co^3+^, and the second doublet was at 781.9 and 797.8 eV, arising from Co 2p_3/2_ and Co 2p_1/2_ of Co^2+^. In addition, two broad peaks located at 803.6 and 786.7 eV are attributed to the satellites, which indicated the presence of Co_3_S_4_ (Xiao et al., 2014; Liu et al., 2015; Gao et al., 2018; Wang X. et al., 2018).

In the Ni 2p spectrum (Figure 4D), there exist two main peaks at 855.7 and 873.5 eV assignable, respectively to Ni 2p_3/2_ and Ni 2p_1/2_ spin orbit doublets and two satellite peaks (862.4 and 880.1 eV). By deconvolution of the two main peaks, the Ni 2p_3/2_ orbit comprises two peaks with binding energy of 853.6 and 856.7 eV, which corresponded, respectively to the Ni^2+^ and Ni^3+^ oxidation states, and the Ni 2p_1/2_ orbit can also be fitted into two peaks belonging to Ni^2+^ (871.5 eV) and Ni^3+^ (875.2 eV) (Hu et al., 2015; Qin et al., 2016; Sivanantham et al., 2016). There show S 2p_3/2_ and S 2p_1/2_ peaks at 161.7 and 162.8 eV in Figure 4E, which can be related to S^2−^ (Wang H. et al., 2018). The component peak at 164.1 eV is characteristics of a metal–sulfur (M-S) bond (Ning et al., 2018). The peaks of 168.9 and 170.1 eV can be attributed to ${\text{SO}}_{4}^{2-}$ due to air oxidation (Cheng et al., 2015). In the O 1s region (Figure 4F), the peaks of 530.0, 531.9, and 533.8 eV are observed on the surface of the catalyst, which are corresponding to O^2−^, hydroxyl group, and adsorbed water molecules, respectively (Luo et al., 2017).

**Figure 5:**
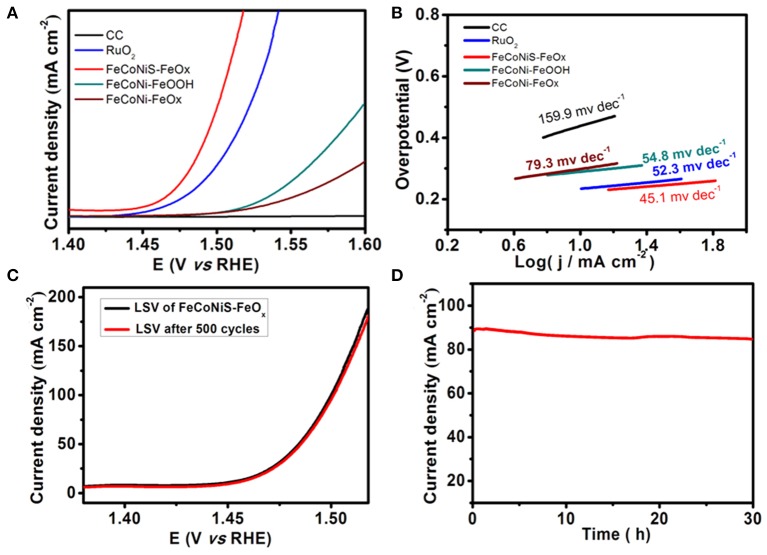
**(A)** Linear sweep voltammetry (LSV) curves of different oxygen evolution reaction (OER) catalysts. **(B)** Tafel curves of different OER catalysts. **(C)** LSV curve of FeCoNiS-FeO_x_ catalyst and another curve after 500 CV cycles. **(D)** Time-dependent current density curve of FeCoNiS-FeO_x_ OER catalyst under constant potential.

The catalysis performance of the catalyst in water oxidation reaction is evaluated by linear sweep voltammetry (LSV), shown in Figure 5A. For comparison, LSV curves of different catalysts with similar loading amounts, including CC, RuO_2_, FeCoNi-FeOOH, FeCoNi-FeO_x_, FeCoNiS-FeO_x_, are also evaluated. Overpotentials in the same current density are often used to estimate the OER performance. FeCoNiS-FeOOH/CC exhibits outstanding OER performance with driving 100 mA cm^−2^ at a low overpotential of 269.9 mV, which is superior to RuO_2_/CC and FeCoNi-FeOOH under the same conditions. FeCoNi-FeO_x_ is prepared through second hydrothermal method without sulfurizing reagent. The OER activity of FeCoNi-FeO_x_ is lower than FeCoNi-FeOOH. The existence of Fe_3_O_4_ will not enhance catalytic reactivity. The main active site of FeCoNiS-FeO_x_ is FeCoNi sulfides. Tafel plots of different OER catalysts were shown in Figure 5B, which are used to evaluate the catalytic kinetics. The Tafel slope of FeCoNiS-FeO_x_ is 45.1 mV dec^−1^, lower than that of RuO_2_/CC (52.3 mV dec^−1^). It demonstrates that the FeCoNiS-FeO_x_ catalyst has more rapid reaction velocity in OER catalytic reaction.

**Figure 6:**
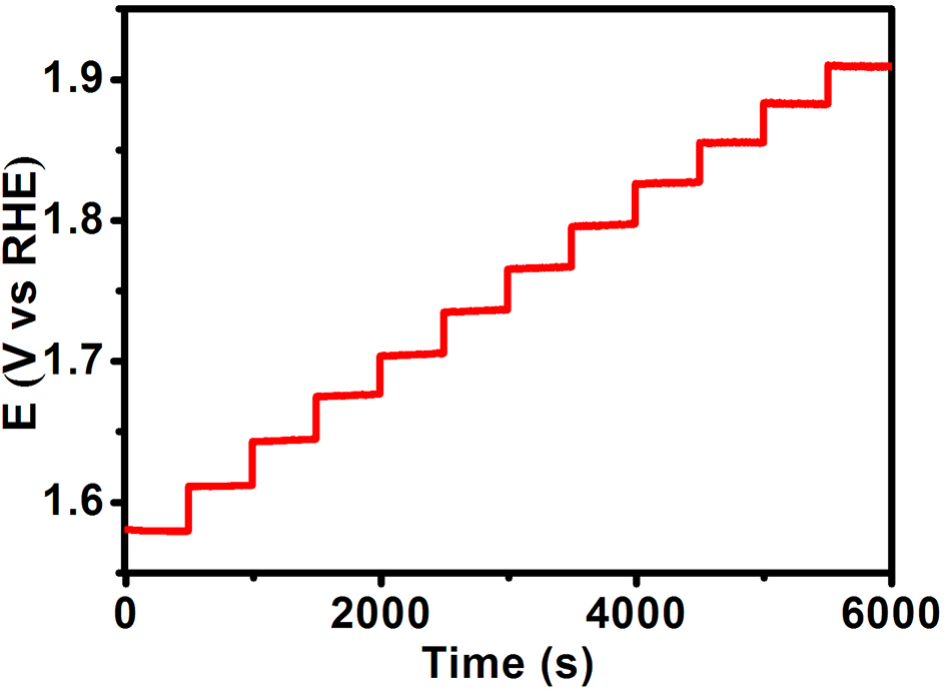
Multistep chronopotentiometric of oxygen evolution reaction (OER) catalyst. The current density started at 60 mA cm^−2^ and finished at 280 mA cm^−2^, with an increment of 20 mA cm^−2^ per 500 s without ohmic potential drop (IR) correction.

Another important performance, stability of catalyst, is investigated by cyclic voltammetry and potentiostatic method. As shown in Figure 5C, there shows a comparison of two polarization curves, including original curve and another curve after 500 CV cycles. When the potential is 1.5 V, the current density is only 3% decrease after 500 cycles, which demonstrates that the FeCoNiS-FeO_x_ catalyst has a good cycle life. In Figure 5D, the electrochemical stability of FeCoNiS-FeOOH/CC is tested by potentiostatic electrolysis at a constant potential of 1.48 V for 30 h. There is only 5% decay of current density, which demonstrated the good long-term durability of the catalyst. Multistep chronopotentiometric curve of FeCoNiS-FeO_x_ is shown in Figure 6. There are 12 steps and the increment of current density is 20 mA cm^−2^ per 500 s. In every step, the corresponding potential remains constant. These results indicate that the catalyst has excellent conductivity and good mass transportation.

## Conclusions

In this paper, a two-step hydrothermal routine is adopted to prepare FeCoNiS-FeO_x_ catalyst. At first, hydroxide nanowire array precursor is prepared. The precursor nanowire arrays serve as backbones for the catalyst not only constructs effective conductive channels but also provides rich active sites. Secondly, the final product is prepared via anion exchange and redox reactions with Na_2_S as sulfurizing reagent. The catalyst shows excellent OER activity needing overpotentials of 220.5 and 269.9 mV to attain current densities of 10 and 100 mA cm^−2^ in 1.0 M KOH. Typically, the catalyst also shows long-term electrochemical stability for at least 30 h. The good catalysis performance is due to FeS, Co_3_S_4_, and Ni_3_S_4_. CC as substrate could enhance the conductivity of the material. Nanowire structure could increase the surface area of materials and expose more active sites. Most importantly, transition-metal sulfide could optimize material structure and give a full play to the synergy effect between different elements. FeCoNiS-FeO_x_ catalyst is a promising electrocatalyst for OER in alkaline media.

## Materials and Methods

### Materials

Ferric nitrate [Fe(NO_3_)_3_.9H_2_O, Mw = 404.00], nickel nitrate [Ni(NO_3_)_2_.6H_2_O, Mw = 290.79], cobalt nitrate [Co(NO_3_)_2_.6H_2_O, Mw = 291.03], ammonium fluoride NH_4_F, Mw = 37.0), urea [CO(NH_2_)_2_, Mw = 60.06], potassium hydroxide (KOH, Mw = 56.1) are provided by Shanghai Aladdin Ltd. Sodium sulfide (Na_2_S, Mw = 78.04) and ruthenium chloride (RuCl_3_.3H_2_O ≥ 43%) are bought from Sigma-Aldrich Co. Ltd. CC is supplied by Jingchong electronics technology company. The surface must be free from oil and dirt, then acetone, hydrochloric acid (3 mol/L), ethanol, and ultrapure water are used to clean the surface of CC. Ultrapure water (18.2 MΩ.cm) is used to prepare all aqueous solutions in this work. None of the reagents as received are further purified.

### Preparation of Precursor

Fe(NO_3_)_3_.9H_2_O 0.323 g, Ni(NO_3_)_2_.6H_2_O 0.058 g, Co(NO_3_)_2_.6H_2_O 0.058 g, NH_4_F 0.03 g, CO(NH_2_)_2_ 0.12 g are added to 20 ml ultrapure water to form mixture solution after 30 min stirring. The final solution and the pretreated CC are sealed in a 30-ml Teflon-lined stainless-steel high-pressure reactor and maintained at 120°C for 5 h. Then, the product is naturally cooled to room temperature. The product is taken from the reactor and washed for three times with ultrapure water and ethanol successively. Dried under 60°C for 2 h.

### Preparation of FeCoNiS-FeO_x_ Nanowire

First, 0.035 g sodium sulfide is added to 20 ml ultrapure water with stirring. The formed solution and the precursor are sealed in a 30-ml Teflon-lined stainless-steel high-pressure reactor and maintain at 120°C for 3 h. After naturally cooling to room temperature, the product is washed with ultrapure water and ethanol successively. At last, the product is dried for 2 h under 60°C.

### Characterizations

A diffractometer (RigakuD/MAX 2550, Cu Kα radiation, λ = 1.5418 Å) is used to perform XRD characterization. The scan range is from 5 to 80° with a scanning rate of 5°/min. SEM characterizations are realized on a MERLIN compact SEM with the accelerating voltage of 20 kV. TEM characterizations are realized on TEM of Zeiss Libra 200FE with operation voltage of 200 kV. An ESCALABMK II X-ray photoelectron spectrometer is used to measure XPS spectrum with Mg as the exciting source.

### Electrochemical Measurements

A CHI 660E electrochemical analyzer (CH Instruments, Inc., Shanghai) is used to perform all the electrochemical tests. In order to better characterize the electrode reaction, a three-electrode system is adopted. The catalysts/CC is used as working electrode. Mercuric oxide electrode (Hg-HgO) is as contrast electrode. Graphite rod is as auxiliary electrode. Potassium hydroxide solution (1.0 M) is used as the working electrolyte solution. All experiments are realized at 25°C. All potentials for LSV curves are calibrated on reversible hydrogen electrode (RHE) scale [E (RHE) = E + 0.059 × 14 + 0.098]. Unless stated otherwise, all LSV potentials are calibrated with ohmic potential drop (IR) due to solution resistance. Overpotentials (ΔE) are calculated based on the equation ΔE = E (RHE) - IR - 1.23.

## Data Availability Statement

All datasets generated for this study are included in the article.

## Author Contributions

WT has done the experimental work. MH, ZS, and WY helped in characterization. XZ has written the manuscript. CD and SZ have revised the manuscript.

## Conflict of Interest

The authors declare that the research was conducted in the absence of any commercial or financial relationships that could be construed as a potential conflict of interest.
